# Inpatient Medicaid Usage and Expenditure Patterns After Changes in Supplemental Nutrition Assistance Program Benefit Levels

**DOI:** 10.5888/pcd15.180185

**Published:** 2018-10-04

**Authors:** Rajan A. Sonik, Susan L. Parish, Monika Mitra

**Affiliations:** 1 Health Equity Research Lab, Cambridge Health Alliance, Harvard Medical School, Cambridge, Massachusetts; 2 Bouvé College of Health Sciences, Northeastern University, Boston, Massachusetts; 3 Lurie Institute for Disability Policy, Heller School for Social Policy and Management, Brandeis University, Waltham, Massachusetts

## Abstract

**Introduction:**

Food insecurity worsens health outcomes and is associated with increased health care usage and expenditures. The Supplemental Nutrition Assistance Program (SNAP) reduces but does not eliminate recipients’ food insecurity. We sought to determine whether inpatient Medicaid usage and expenditure patterns responded to an April 2009 increase in SNAP benefit levels and a subsequent November 2013 decrease.

**Methods:**

Interrupted time series models estimated responses to the 2009 and 2013 SNAP changes in the Medicaid population, compared responses between Medicaid and Medicare recipients, and compared responses between Medicaid recipients with different likelihoods of having a disability. Analyses used 2006 through 2014 Healthcare Cost and Utilization Project National (previously Nationwide) Inpatient Sample data.

**Results:**

After the 2009 SNAP increase, Medicaid admission growth fell nationally from 0.80 to 0.35 percentage points per month (a difference of –0.45; 95% CI, –0.72 to –0.19), adjusting for enrollment. After the 2013 SNAP decrease, admission growth rose to 2.42 percentage points per month (a difference of 2.07; 95% CI, 0.68 to 3.46). Inflation-adjusted monthly Medicaid expenditures followed similar patterns and were associated with $26.5 billion (in 2006 dollars) in reduced expenditures over the 55 months of the SNAP increase, and $6.4 billion (in 2006 dollars) in additional expenditures over the first 14 months after the SNAP decrease. Effects were elevated for Medicaid compared with Medicare recipients and among people with a high likelihood of having a disability.

**Conclusion:**

Although alternative causal explanations warrant consideration, changes in SNAP benefit levels were associated with changes in inpatient Medicaid usage and cost patterns.

## Introduction

Food insecurity is a determinant of population health (1) associated with multiple health problems (2) and elevated health care usage and expenditures (3). In 2016, 12% of US households experienced food insecurity (4). The Supplemental Nutrition Assistance Program (SNAP), serving 44 million Americans in 2016 (5), partially alleviates recipients’ food insecurity (6). It follows that fluctuations in SNAP benefit levels may affect food insecurity and in turn health care usage and expenditures. Conceptual models for these hypotheses propose that food insecurity increases the risk of developing chronic diseases in the long term and exacerbating these conditions in the short term (7). These effects occur via skipped meals (8), poor nutrition quality (9), stress and depression (10), impaired decision-making capacity (11), and tradeoffs with key resources such as medication and housing (11,12).

Recent policy changes provide a unique opportunity for studying these pathways. The American Recovery and Reinvestment Act increased monthly SNAP benefits by a minimum of 13.6% per SNAP household in April 2009 (13), and this increase expired in November 2013 (13). Both the 2009 and 2013 changes were immediate and affected all recipients across the United States. Moreover, the post–Great Recession recovery did not reach those eligible for SNAP during much of this time period, leading to a poor but stable economic environment; wealth grew for higher-income families during this period but was stagnant for lower-income families (14).

One study of the 2009 SNAP increase found it was associated with decreased growth in inpatient Medicaid admissions and expenditures in Massachusetts, particularly among people with chronic illnesses (15). Another report examining both the 2009 and 2013 SNAP changes found no effect on health outcomes, though it did not adjust for enrollment and used limited data points (16). Our objective was to build on these approaches by examining changes in nationwide usage, expenditure, and enrollment data at multiple time points. We compared effects among Medicaid and Medicare populations and among recipients with varying likelihoods of having a disability. Effects should be greater in Medicaid versus Medicare, because Medicaid recipients have greater exposure to SNAP benefits (13). Effects of SNAP should also be magnified for people with disabilities, who have elevated exposure to food insecurity and elevated SNAP and health care usage (17,18).

## Methods

### Data

We used data from the 2006 through 2014 Healthcare Cost and Utilization Project’s Nationwide Inpatient Sample (renamed National Inpatient Sample in 2012), which provide all-payer inpatient discharge data covering of 97% of the United States population (19), including detailed medical, expenditure, and demographic data for each hospital admission (19). The Inpatient Sample was redesigned in 2012; however, by using “trend weights” for years before 2012 and original weights for later years, nationally representative trends spanning periods before and after 2012 can be calculated (19).

### Analytic samples

Three analytic samples were identified. In the first, data from discharges for which the primary payer was Medicaid were collapsed by month. This made the 108 months from January 2006 through December 2014 the units of observations. Summary data for each month (eg, total admissions) were included as model covariates. This sample allowed for interrupted time series analyses examining the Medicaid population as a whole.

For the second analytic sample, we replicated the procedures used for the overall Medicaid sample with the Medicare sample and then combined them. The payer was assigned based on the primary payer listed, which was typically Medicare for inpatient stays for dual eligible individuals. Summary data for each payer for each month were again the covariates, in addition to a variable identifying the payer for each observation. This sample allowed for analyses comparing the Medicaid and Medicare populations.

For the final analytic sample, each Medicaid discharge was identified as being for someone with no, low, moderate, or high likelihood of having a disability. To identify disability likelihood, we used a modified version (20) of the Access Risk Classification System (version 2) algorithm (21), which uses information from *International Classification of Diseases, Ninth Revision, Clinical Modification*, codes and Healthcare Common Procedure Coding System codes.

This modified algorithm has a sensitivity for identifying people with disabilities of 49% to 83% and a specificity of 30% to 80% when dichotomizing individuals into no/low and moderate/high disability likelihood categories (20,21). To increase the specificity of this algorithm, as done previously (22), we identified the individuals with the highest likelihood of having a disability and compared them to the other groups. After each discharged person was assigned a likelihood for having a disability, data for each group was collapsed by month. Consequently, each month had 4 observations, 1 for each group (no, low, moderate, and high). Summary data for each group for each month were the covariates in addition to a variable identifying the likelihood group for each observation. This sample allowed for analyses comparing the different disability likelihood subsets of the Medicaid population.

### Dependent variables

We used 3 dependent variables: total monthly admissions, monthly average length of stay per admission, and total monthly inflation-adjusted inpatient costs. When collapsing monthly data, as in the prior study examining effects of the 2009 SNAP increase in Massachusetts (15), we generated the total number of admissions (the weighted sum of the number of discharges) and the weighted average length of stay per admission. Before totaling monthly costs, we multiplied hospital charges for each admission by the cost-to-charge ratio provided by the National Inpatient Sample (23). Hospitals charge differently for similar procedures, so each has a different cost-to-charge ratio. Multiplying charges by cost-to-charge ratios yielded actual costs (23). We used the provided weights to produce nationally representative estimates (trend weights for the 2006–2011 data and original weights for the 2012–2014 data) (19). Weighted total cost figures were then summed to obtain total monthly costs.

Medical care inflation was significantly higher than general inflation during the study period (24), but Medicaid inflation was also lower than overall medical inflation (25). To estimate Medicaid-specific inflation and generate inflation-adjusted costs, we calculated the monthly change in the average cost per day of admission. Because this inflation measure was internal to the data, we could estimate inflation figures specific to Medicaid, to the different disability likelihood groups, and to Medicare.

As was done in the Massachusetts study (15), we scaled each dependent variable to be the percentage change from the value in the first month, January 2006. This scaling standardized interpretations across dependent variables and comparison groups.

### Covariates

For covariates, weighted monthly demographic figures were calculated. These figures included the average age of patients and the percentages of discharges for which the individual discharged was female, non-Hispanic white, or lived in a zip code with a median annual income in the lowest quartile (below $36,000 to $39,000, depending on the year). Enrollment per month was another covariate. Medicaid and Medicare enrollment numbers were available for June of each year of the study period through 2013 and on a monthly basis for 2014 (26,27). Enrollment numbers for months other than June for 2006 through 2013 were projected using linear interpolation based on the available numbers. Enrollment was not a covariate when comparing disability likelihood groups because such numbers are not available by disability likelihood.

We also created variables for the purposes of the interrupted time series approach. First was a “month” counter variable, with a value of 1 for January 2006 and 108 for December 2014. The interruption points, which each had dichotomous post variables and postcounter variables, were April 2009 (month 40) and November 2013 (month 95), coinciding with the increase and subsequent decrease in SNAP benefits. For the Medicare comparison, there was a dichotomous payer variable and interaction terms between this variable and all time-related variables. Similarly, for the disability analyses, there was also a dichotomous disability variable and interaction terms between this variable and all time-related variables.

### Statistical analysis

Single-group interrupted time series models test for 2 potential data pattern changes after a policy change (“interruption”): changes in trends (slope) and immediate changes in level (intercept) (28). Multiple interruptions can be tested in 1 model (28). Multigroup interrupted analyses are similar, but they also allow for comparisons of how the patterns of different groups change after interruptions (28). Inferences from multigroup analyses are thus analogous to those from difference-in-differences models. The statistical models used here are provided in the Appendix. We used Stata software, version 14.0 (StataCorp LLC), and its ITSA package to account for autocorrelation and heteroskedasticity via Newly-West standard errors. We allowed consideration of up to 12 months of lag in the autocorrelation structure.

## Results

### Medicaid population as a whole

During January 2006 through March 2009, adjusting for enrollment and other covariates, the number of monthly Medicaid admissions across the United States rose by an average of 0.80 percentage points per month over the baseline January 2006 number (Table 1). This growth slowed to 0.35 percentage points per month (a difference of –0.45; 95% CI, –0.72 to –0.19) from April 2009 to October 2013, and then rose again to 2.42 percentage points per month (a difference of 2.07; 95% CI, 0.68 to 3.46) starting in November 2013 (Table 1). Monthly inpatient Medicaid expenditures followed a similar pattern: initial growth of 0.85 percentage points per month, a slowdown in growth to 0.36 percentage points per month (a difference of –0.49; 95% CI, –0.73 to –0.25) after the April 2009 SNAP increase, and a subsequent increase in this growth to 2.09 percentage points per month (1.73; 95% CI, 0.37 to 3.09) after the November 2013 SNAP decrease (Table 1, Figure). The changes in expenditure patterns were associated with a total savings of $26,465,103,280 (in January 2006 dollars) over the 55 months of the SNAP increase and a total cost of $6,374,326,245 (in January 2006 dollars) over the first 14 months after the SNAP decrease. The immediate changes in overall levels of monthly admissions and expenditures after the SNAP policy changes were not significant. Patterns for the average length of stay differed, because they did not change significantly after the April 2009 SNAP increase. After the November 2013 SNAP decrease, there was an immediate 1.33 percentage point increase (95% CI, 0.40 to 2.26) in the average length of stay, but this was offset over time by a slowdown in growth of –0.30 percentage points per month (95% CI, –0.55 to –0.05) (Table 1).

**Table 1:** Changes in Nationwide Inpatient Medicaid Trends in Response to an April 2009 Increase and November 2013 Decrease in SNAP Benefit Levels, 2006–2014^a^

Category	Monthly Admissions in Percentage Points^b^ (95% CI)	Monthly Expenditures^c^ in Percentage Points^b^ (95% CI)	Monthly Average Length of Stay per Admission in Percentage Points^b^ (95% CI)
**Variable**			
Change in percentage points^d^ per month before SNAP increase	0.80^e^ (0.31 to 1.29)	0.85^e^ (0.36 to 1.34)	0.03 (−0.05 to 0.11)
2009 SNAP increase, immediate change in level that month	11.17 (−0.02 to 22.36)	9.86 (−1.45 to 21.17)	−0.99 (−2.46 to 0.49)
Change in slope^d^ after SNAP increase	−0.45^e^ (−0.72 to −0.19)	−0.49^f^ (−0.73 to −0.25)	−0.02 (−0.07 to 0.02)
2013 SNAP decrease, immediate change in level that month	−3.90 (−11.87 to 4.06)	−2.12 (−9.69 to 5.44)	1.33^e^ (0.40 to 2.26)
Change in slope^d^ after SNAP decrease	2.07^e^ (0.68 to 3.46)	1.73^g^ (0.37 to 3.09)	−0.30^g^ (−0.55 to −0.05)
**Covariates**			
Medicaid enrollment, in millions	−1.81 (−3.92 to 0.31)	−1.94 (−4.13 to 0.25)	−0.04 (−0.47 to 0.38)
Percentage of female admissions	−1.85 (−4.70 to 1.01)	−3.28^g^ (−6.10 to −0.47)	−1.17^f^ (−1.60 to −0.74)
Percentage of non-Hispanic white admissions	0.27 (−1.38 to 1.92)	0.02 (−1.54 to 1.58)	−0.21^g^ (−0.37 to −0.05)
Percentage of lowest income quartile admissions^h^	3.78^f^ (1.89 to 5.67)	3.70^e^ (1.66 to 5.75)	−0.03 (−0.38 to 0.33)
Average age, y	−1.70 (−4.22 to 0.83)	−0.57 (−2.94 to 1.79)	0.90^f^ (0.49 to 1.31)

Abbreviations: CI, confidence interval; SNAP, Supplemental Nutrition Assistance Program.

a Single group interrupted time series models using Newey-West standard errors (constant omitted).

b All dependent variables scaled by dividing by the value in January 2006, subtracting 1, and multiplying by 100 (this produces coefficients that can be read as percentage points of the January 2006 value).

c Monthly expenditures adjusted for inflation.

d Slope can be interpreted as the changes in percentage points per month (for example, a coefficient of 2 on a slope term would indicate a change in 2 percentage points per month; this would mean that after 3 months the value would have increased by 6% of the January 2006 value).

e *P* < .01.

f *P* < .001.

g *P* < .05.

h This was equivalent to annual income less than $36,000 to $39,000, depending on the year.

**Figure:**
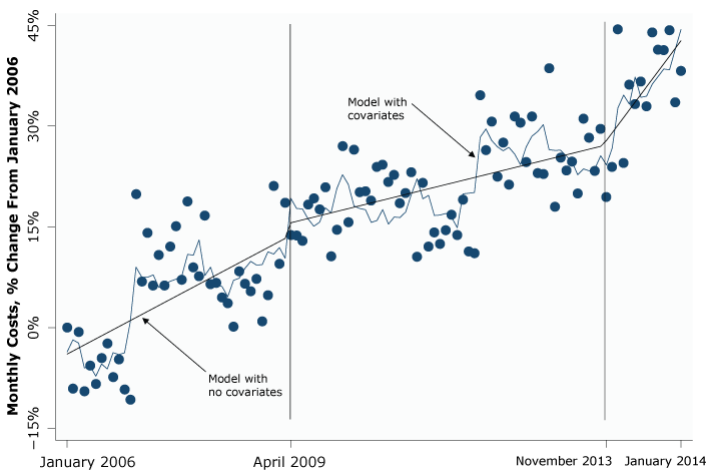
Interrupted time series analysis of changes in nationwide inpatient Medicare expenditures in response to changes in the Supplemental Nutrition Assistance Program (SNAP), January 2006–January 2014. SNAP benefits increased monthly by a minimum of 13.6% per SNAP household in April 2009, and this increase expired in November 2013.

### Medicaid-Medicare comparison

Before the April 2009 SNAP increase, expenditure growth did not differ significantly between the 2 groups (Table 2). After the April 2009 SNAP increase, admission growth slowed significantly more for Medicaid than for Medicare (–0.26 percentage points per month; 95% CI, –0.48 to –0.04), though Medicaid also had a larger immediate change at the time (22.30 percentage points; 95% CI, 8.43 to 36.18) (Table 2). After the November 2013 SNAP decrease, admission growth increased significantly more for Medicaid than for Medicare (2.26 percentage points per month; 95% CI, 0.84 to 3.67) (Table 2). Differences in Medicaid and Medicare expenditure patterns paralleled the differences in admission patterns, though differences in average length of stay patterns were minimal (Table 2).

**Table 2:** Comparison of Nationwide Inpatient Medicaid and Medicare Trends Before and After an April 2009 Increase and November 2013 Decrease in SNAP Benefit Levels, 2006–2014^a^

Variable	Monthly Admissions in Percentage Points^b^ (95% CI)	Monthly Expenditures^c^ in Percentage Points^b^ (95% CI)	Monthly Average Length of Stay per Admission in Percentage Points^b^ (95% CI)
**Medicare**			
Change in percentage points^d^ per month before SNAP increase	0.65^e^ (0.41 to 0.88)	0.59^e^ (0.36 to 0.82)	−0.05^f^ (−0.09 to 0)
2009 SNAP increase, immediate change in level that month	−8.5^g^ (−14.23 to −2.77)	−10.42^e^ (−15.97 to −4.87)	−1.99^e^ (−2.78 to −1.21)
Change in slope^d^ after SNAP increase	−0.14 (−0.38 to 0.11)	−0.16 (−0.40 to 0.09)	0 (−0.03 to 0.03)
2013 SNAP decrease, immediate change in level that month	−3.31 (−6.73 to 0.11)	0.75 (−2.95 to 4.45)	2.99^e^ (2.16 to 3.83)
Change in slope^d^ after SNAP decrease	−0.14 (−0.47 to 0.18)	−0.24 (−0.57 to 0.09)	−0.09 (−0.20 to 0.01)
**Medicaid**			
Difference in slope^d^ before SNAP increase	0.08 (−0.25 to 0.40)	0.23 (−0.08 to 0.54)	0.12^e^ (0.07 to 0.17)
Difference in immediate change^g^ in level for the month of the 2009 SNAP increase	22.30^h^ (8.43 to 36.18)	22.64^h^ (8.66 to 36.62)	0.88 (−0.68 to 2.44)
Difference in change in slope^d^^,^ g after SNAP increase	−0.26^f^ (−0.48 to −0.04)	−0.29^f^ (−0.52 to −0.07)	−0.05 (−0.11 to 0)
Difference in immediate change in level for the month of the 2013 SNAP decrease	−0.29 (−7.15 to 6.57)	−3.18 (−9.27 to 2.90)	−2.24^f^ (−4.01 to −0.47)
Difference in change in slope^d^^,^ g after SNAP decrease	2.26^h^ (0.84 to 3.67)	2.07^h^ (0.69 to 3.44)	−0.16 (−0.46 to 0.13)
**Covariates**			
Medicaid enrollment, in millions	−1.94 (−4.27 to 0.40)	−2.27 (−4.54 to 0)	−0.24 (−0.64 to 0.15)
Percentage of female admissions	−0.23 (−3.56 to 3.10)	−1.87 (−5.19 to 1.44)	−1.36^e^ (−1.79 to −0.93)
Percentage of non-Hispanic white admissions	0.81 (−1.00 to 2.62)	0.55 (−1.22 to 2.31)	−0.20^f^ (−0.36 to −0.05)
Percentage of lowest income quartile admissions^i^	3.98^e^ (2.68 to 5.28)	3.87^e^ (2.49 to 5.25)	0.05 (−0.18 to 0.28)
Average age, y	0.07 (−2.55 to 2.68)	1.63 (−1.03 to 4.29)	1.4^e^ (0.89 to 1.99)

Abbreviations: CI, confidence interval; SNAP, Supplemental Nutrition Assistance Program.

a Multigroup interrupted time series models using Newey-West standard errors (constant and term comparing initial intercept between groups omitted).

b Dependent variable scaled by dividing by the value in January 2006, subtracting 1, and multiplying by 100 (this produces coefficients that can be read as percentage points of the January 2006 value).

c Monthly expenditures adjusted for inflation.

d Slope can be interpreted as the changes in percentage points per month (for example, a coefficient of 2 on a slope term would indicate a change in 2 percentage points per month; this would mean that after 3 months the value would have increased by 6% of the January, 2006 value).

e *P* < .001.

f *P* < .05.

g All “difference in change” terms should be interpreted as difference-in-differences terms (for example, a difference in change in slope indicates how the change in the slope for the Medicaid population differed from the change in slope for the Medicare population).

h *P* < .01.

i This was equivalent to annual income less than $36,000 to $39,000, depending on the year.

### Comparing disability likelihood groups

Differences in admission patterns between the group with a high likelihood of having a disability and the group with no likelihood of having a disability were in the hypothesized directions but not significant (Table 3), although differences in admission patterns between the high likelihood and the low likelihood groups and the high likelihood and moderate likelihood groups were significant. Expenditure results also were in the hypothesized directions and varied in terms of significance across comparisons. Before the April 2009 SNAP increase, expenditures for the high likelihood group rose faster than they did for all other groups. Greater expenditure slowdowns for the high versus no groups after the April 2009 SNAP increase were not significant (Table 3), but they were in the comparisons of the high versus low groups and the high versus moderate groups. After the November 2013 SNAP decrease, expenditures for the high group had a larger immediate jump than both the no group (Table 3) and the low group, but not the moderate group. Results for average length of stay were less consistent in terms of direction and significance.

**Table 3:** Comparison of Nationwide Inpatient Medicaid Trends Among Individuals With No Likelihood of Having a Disability and With High Likelihood of Having a Disability Before and After an April 2009 Increase and November 2013 Decrease in SNAP Benefit Levels, 2006–2014^a^

Variable	Monthly Admissions in Percentage Points^b^ (95% CI)	Monthly Expenditures^c^ in Percentage Points^b^ (95% CI)	Monthly Average Length of Stay per Admission in Percentage Points^b^ (95% CI)
**No likelihood of having a disability**			
Change in percentage points^d^ per month before SNAP increase	0.41^e^ (0.13 to 0.69)	0.33^f^ (0.07 to 0.58)	−0.08^e^ (−0.12 to −0.03)
2009 SNAP increase, immediate change in level that month	4.33 (−3.28 to 11.94)	3.36 (−4.00 to 10.72)	−0.40 (−1.67 to 0.88)
Change in slope^d^ after SNAP increase	−0.61^g^ (−0.94 to −0.29)	−0.50^e^ (−0.79 to −0.21)	0.09^g^ (0.05 to 0.14)
2013 SNAP decrease, immediate change in level that month	−4.05 (−8.77 to 0.67)	−2.96 (−7.30 to 1.37)	0.92^f^ (0.08 to 1.76)
Change in slope^d^ after SNAP decrease	0.70^e^ (0.26 to 1.15)	0.57^e^ (0.15 to 0.99)	−0.09^f^ (−0.16 to −0.02)
**High likelihood of having a disability**			
Difference in slope^d^ before SNAP increase	0.33 (−0.05 to 0.71)	0.36^f^ (0.02 to 0.70)	0.03 (−0.01 to 0.08)
Difference in immediate change^h^ in level for the month of the 2009 SNAP increase	6.00 (−6.19 to 18.19)	3.35 (−8.12 to 14.82)	−2.10^e^ (−3.33 to −0.87)
Difference in change in slope^d^^,^ h after SNAP increase	0.26 (−0.20 to 0.71)	0.06 (−0.35 to 0.48)	−0.13^g^ (−0.18 to −0.07)
Difference in immediate change in level for the month of the 2013 SNAP decrease	8.71 (−2.14 to 19.56)	9.68^f^ (0.68 to 18.68)	0.41 (−1.38 to 2.19)
Difference in change in slope^d^^,^ h after SNAP decrease	1.00 (−0.20 to 2.20)	0.45 (−0.45 to 1.34)	−0.24^f^ (−0.45 to −0.03)
**Covariates**			
Percentage of female admissions	1.36 (−1.77 to 4.49)	0.28 (−2.69 to 3.26)	−0.91^e^ (−1.29 to −0.53)
Percentage of non-Hispanic white admissions	0.11 (−1.23 to 1.46)	−0.04 (−1.29 to 1.21)	−0.10 (−0.27 to 0.07)
Percentage of lowest income quartile admissions^i^	2.81^f^ (0.50 to 5.11)	2.57^f^ (0.42 to 4.71)	−0.10 (−0.35 to 0.15)
Average age, y	−2.80 (−8.20 to 2.60)	−1.61 (−6.46 to 3.25)	0.84^f^ (0.14 to 1.53)

Abbreviations: CI, confidence interval; SNAP, Supplemental Nutrition Assistance Program.

a Multigroup interrupted time series models using Newey-West standard errors (constant and term comparing initial intercept between groups omitted).

b Dependent variable scaled by dividing by the value in January 2006, subtracting 1, and multiplying by 100 (this produces coefficients that can be read as percentage points of the January 2006 value).

c Monthly expenditures adjusted for inflation.

d Slope can be interpreted as the changes in percentage points per month (for example, a coefficient of 2 on a slope term would indicate a change in 2 percentage points per month; this would mean that after 3 months the value would have increased by 6% of the January, 2006 value).

e *P* < .01.

f *P* < .05.

g *P* < .001.

h All “difference in change” terms should be interpreted as difference-in-differences terms (for example, a difference in change in slope indicates how the change in the slope for the group with a high likelihood of disability differed from the change in slope for the group with no likelihood of disability).

i This was equivalent to annual income less than $36,000 to $39,000, depending on the year.

## Discussion

In the Medicaid population, monthly hospital admissions were increasing from January 2006 until the April 2009 SNAP increase. The rate of this growth fell significantly after the April 2009 SNAP increase and rose significantly after the November 2013 SNAP decrease. Expenditure patterns matched admission patterns closely and were associated with $26.5 billion in savings over the 55 months of the SNAP decrease and $6.4 billion in added costs during the first 14 months of the SNAP decrease. Cost growth slowed more after April 2009 and increased more after November 2013 for the Medicaid population than it did for the Medicare population, whose recipients have less exposure to SNAP. This difference indicates an effect beyond general health care patterns. Further, using a rough identifier of disability likelihood, moderate evidence suggested that Medicaid recipients with a high likelihood of having a disability, a group with greater food insecurity exposure and sensitivity, were more responsive to SNAP changes than were Medicaid recipients with a lower likelihood of having a disability. Interrupted time series models involving a comparison group and both the introduction and removal of a policy are among the most robust quasi-experimental designs (28). These findings thus offer support for the hypothesis that inpatient Medicaid cost and usage patterns are responsive to changes in SNAP benefit levels.

Several limitations must be considered. SNAP and Medicaid populations do not overlap perfectly. Many Medicaid recipients do not receive SNAP benefits, meaning they would not have been affected by changes in SNAP benefit levels. This likely made the results conservative, however, because it reduced our ability to detect the effects of the SNAP changes precisely. Another potential limitation was the use of internal inflation data. By using the average costs per day of admission, we may have captured both changes in inpatient Medicaid inflation and changes in the quantity and intensity of services provided per day. If so, and if fluctuations in the daily quantity and intensity of services differed substantially from changing patterns in admissions and lengths of stay, then these results could have been biased. This possibility was likely small, as the inflation figures used here were consistent with previous Medicaid inflation research (25), and the lack of changes in length of stay per admission do not suggest changes in case mix. Separately, results were limited to inpatient data and could be offset by changes in other types of care that were not measured. Additionally, although the comparison to Medicare data was a strength, further studies with more detailed claims data might allow for more nuanced comparisons with additional payers. Privately insured individuals are even less likely to receive SNAP than Medicare recipients are, but they made for a less feasible comparison here because of large changes in private insurance markets during the study period. Finally, the study period included multiple other policy changes, including at the state level, that we could not control for here given the lack of state indicators in recent Healthcare Cost and Utilization Project inpatient sample data. However, the immediacy and uniformity of the SNAP changes and the general lack of economic growth for SNAP-eligible populations during the study period (14) offer some buffering against this limitation.

Increases in admissions and expenditure trends after the November 2013 SNAP decrease were markedly larger than the decreases following the April 2009 SNAP increase, despite the 2009 change being larger (the expiration of the SNAP increase coincided with a cost-of-living adjustment, partially offsetting the decrease [13]). Although we adjusted for Medicaid enrollment, state Medicaid expansions starting in 2014 could explain part of the difference if the expansion population had significantly greater medical needs than the pre-expansion population. Pent-up demand is also possible, though it would be unlikely to fully explain the large change in cost and admission patterns, especially given the higher income of the expansion population. Another possibility is that people may be more sensitive to increases in food insecurity than to alleviations of food insecurity. This would be consistent with concepts from ecosocial theory positing that harms can build up in people’s bodies over time and may be easier to exacerbate than to expunge (29). If true, food benefit cuts may lead to larger health effects than increases in food benefits. Further examination of these alternatives is warranted.

Findings from the multigroup models comparing those with high likelihood of disability to other groups were less uniform, though still broadly consistent with the hypothesis that people with disabilities are especially responsive to changes in SNAP benefit levels. One potential limitation to clearer findings was the low specificity of the Access Risk Classification System (version 2) algorithm. It is unlikely that this low specificity differentially affected groups with different sensitivities to changes in SNAP benefits, however, so the resulting misclassification bias was likely nondifferential and made our results more conservative. Another potential explanation is the large diversity within the disability population. Parts of this population may be less sensitive to changes in food security than others. If true, broad comparisons between those with and without disabilities would yield weaker associations. Study of more nuanced stratifications of the disability population may clarify this issue.

Although alternative causal explanations warrant consideration, particularly those related to state policy changes that could not be examined here, our findings overall suggest that proposed cuts to the SNAP program (30) may increase Medicaid usage and expenditures. Proposed Medicaid coverage cuts would offset Medicaid-specific costs, but such cuts will likely amplify associated negative health effects. Moreover, if low-income people experience worsening health and reduced health care access simultaneously, burdens on hospitals may rise if they are forced to provide more unreimbursed emergency care. Financial costs for the health care system may rise overall as a result, even if Medicaid-specific costs are lowered.

The public health consensus is that social factors drive health outcomes (1), but few studies have explored the effects on health care of alleviating or exacerbating social ills such as hunger. Our findings suggest that health care usage and expenditures may be responsive to changes in certain social policies.

## Appendix

The single-group and multigroup interrupted times series models used in this study were as follows:

**Single-group**

Admissions_t_ = β_0_ + β_1_ × (Months)_t_ + β_2 _× (Post_April_2009)_t_ + β_3_ × (Months_Post_April_2009)_t_

+ β_4_ × (Post_November_2013)_t_ + β_5_ × (Months_Post_November_2013)_t_ + β_6–9_ × (Covariates)

+ e_t_

**Multigroup**

Admissions_t_ = β_0_ + β_1_ × (Months)_t_ + β_2_ × (Post_April_2009)_t_ + β_3_ × (Months_Post_April_2009)_t_

+ β_4_ × (Post_November_2013)_t_ + β_5_ × (Months_Post_November_2013)_t_ + β_6_ × (Disability)

+ β_7_ × (Months) × (Disability)_t_ + β_8_ × (Post_April_2009) × (Disability)_t_

+ β_9_ × (Months_Post_April_2009) × (Disability)_t_ + β_10_ × (Post_November_2013) × (Disability)_t_

+ β_11_ × (Months_Post_November_2013) × (Disability)_t_ + β_12–15_ × (Covariates) + e_t_

The coefficients of interest for testing the research hypotheses described in the text were β_2_ through β_5_ in the single-group models and β_7_ through β_11_ in the multigroup models.

The dependent variable listed in the models is admissions, but the same model formulations were used for expenditures and average length of stay. Also, the comparison group listed is disability, but the same model formulations were used for the Medicaid-Medicare comparisons.
